# Comparison of genotype- and haplotype-based approaches for fine-mapping of alcohol dependence using COGA data

**DOI:** 10.1186/1471-2156-6-S1-S65

**Published:** 2005-12-30

**Authors:** Dushanthi Pinnaduwage, Laurent Briollais

**Affiliations:** 1 Division of Epidemiology and Biostatistics, Samuel Lunenfeld Research Institute, Mount Sinai Hospital, Toronto, Ontario, M5G 1X5, Canada; 2 Litwin Centre for Cancer Genetics, and Samuel Lunenfeld Research Institute, Mount Sinai Hospital, Toronto, Ontario, M5G 1X8, Canada; 3 Department of Public Health Sciences, University of Toronto, Toronto, Ontario, Canada

## Abstract

It is generally assumed that the detection of disease susceptibility genes via fine-mapping association study is facilitated by consideration of marker haplotypes. In this study, we compared the performance of genotype-based and haplotype-based association studies using the Collaborative Study of Genetics of Alcoholism dataset, on several chromosomal regions showing evidence for linkage with ALDX1. After correction for multiple testing, the most significant results were observed with the genotype-based analyses on two regions of chromosomes 2 and 7. Interestingly, the analyses results from this dataset showed that there was no advantage of the haplotype-based analyses over genotype-based (single-locus) analyses. However, caution should be taken when generalizing these results to other chromosomal regions or to other populations.

## Background

In fine-mapping of complex diseases, it is generally assumed that the detection of susceptibility genes is facilitated by consideration of marker haplotypes.

Haplotype-based association methods could be more powerful because they capture available linkage disequilibrium (LD) information, but some lack of efficiency could result from the resolution of the gametic phase. When we have many loci to consider, as in the case in fine-mapping of small chromosomal regions, the number of possible haplotypes and degrees of freedoms increase exponentially. On the other hand, the power of genotype-based association test may suffer because LD information contained in flanking markers is ignored.

In this study, we compared the performance of the genotype-based and the haplotype-based association analysis using the Collaborative Study of Genetics of Alcoholism (COGA) dataset, as released through the Genetic Analysis Workshop 14 (GAW14). A large collection of families containing multiple alcohol-dependent (AD) individuals was systematically ascertained by the COGA through probands treated for AD and having at least two other first-degree relatives with AD. It is estimated that 50 to 60% of the variance in AD can be attributed to genetic factors [1,2] and is likely that alcoholism is a complex genetic disorder that results from the action of multiple genes and environmental influences [3,4]. Further, some studies have shown evidence of linkage of AD to some regions on chromosome 1, 2, 4, 7, and 11 [3-5]. In our study, we first carried out a whole-genome scan to identify chromosomal regions linked to AD and then used a fine-scale mapping on the significant regions by genotype- and haplotype-based association tests. The relative advantages of each approach are discussed in light of our analyses of the COGA data.

## Methods

### Data

The COGA data provided by GAW14 includes 143 families with 1,614 individuals representing multiple ethnicities. The data include information on family relationships, discrete and quantitative phenotypes, covariates and genome-wide microsatellite genotypes and single-nucleotide polymorphism (SNP) genotypes from Affymetrix and Illumina as well as genetic maps for both microsatellite and SNPs. We used the disease classification, ALDX1 (AD based on the DSM-III-R and Feighner definition), as the phenotype for genome scan and fine mapping. ALDX1 is coded in five classes: pure unaffected, never drank, unaffected with some symptoms, affected, and unknown. We use a broad definition of ALDX1 by combining the first three codings as unaffected.

### Heritability and genome-wide linkage analysis

First, heritability estimates conditional on the proband's status were computed using the program SOLAR [6]. Then we conducted a preliminary linkage genome scan to identify the chromosomal regions linked to ALDX1. We used these regions for fine-mapping and compared genotype- and haplotype-based association tests. A multipoint genome scan with microsatellite markers was performed at each marker, using the nonparametric linkage approach implemented in the program MERLIN [7] with ALDX1. Genetic map distances were converted from Kosambi to Haldane centimorgans as needed by MERLIN. Nine large families were trimmed manually, leaving a total of 1,551 family members for the genome scan. Allele frequencies were estimated using founders. Scans were performed using nonparametric linkage (NPL) S_pair _and S_all _statistics [8]. COGA families represent multiple ethnicities that could be genetically heterogeneous. Thus, we performed genome scan analyses on the whole sample and on Whites only (115 out of 143 families had a Whites proband).

### Fine-scale association mapping

To narrow down the candidate region showing the strongest evidence for linkage, we performed a fine-scale mapping approach using family-based association tests (FBAT) [9]. We used SNPs and the SNP map given by Affymetrix for the fine mapping in the regions identified by multipoint genome scan. We used all families since FBATs are robust to population admixture. Alleles transmitted to affected offspring are compared with the expected distribution of alleles among offspring. The method is implemented in the software FBAT [10]. The general "FBAT" statistic is based on a linear combination of offspring genotypes and traits:



in which *X*_*ij *_denotes some function of the genotype *G*_*ij *_of the *j*^th ^offspring in family *i *at the locus being tested. *T*_*ij *_is some function of the trait, depending upon possibly unknown parameters. Two different approaches were used based on either unphased genotype information (genotype-based) or haplotypes inferred from genotypes (haplotype-based). In the first approach, the FBAT test statistic is based on the distribution of the offspring genotypes conditional on any trait information and on the parental genotypes. In the second, offspring genotype patterns *G*_*ij *_are phase-known and the genotype coding *X*(*G*_*ij*_) is defined as[11]:



where *k *sums over the set of possible phased genotypes that are compatible with *G*_*ij*_. The sum of weights  over *k *equals 1. The weights are estimated by the conditional probability of observing the phased genotype conditional on the observed unphased genotype. Details about the expectation maximization (EM) algorithm are given in Horvath et al. [11]. Because we tested association in the presence of linkage and the sample consists of family data, a robust variance estimator for *S *was used [12]. Large families were broken into all possible nuclear families, and their contribution to the test statistic was evaluated independently.

Haplotypes were constructed using a 3-locus sliding-window. Two types of tests were performed: a biallelic test (with 1 df) in which each haplotype is tested individually and a multiallelic test in which the degrees of freedoms equal the number of haplotypes minus one. Single-marker tests of association (genotype-based) were carried out in the presence of linkage under an additive model. To correct for multiple testing, the false discovery rate (FDR) principle [13] was applied. Further, for each 3-SNPs window, we calculated the mean LD (i.e., mean of pair-wise LD between adjacent SNPs). LD was measured using Lewontin's D' as estimated by the program GOLD [14].

## Results

### Heritability estimates and genome-wide multipoint linkage analysis

**Figure 1:**
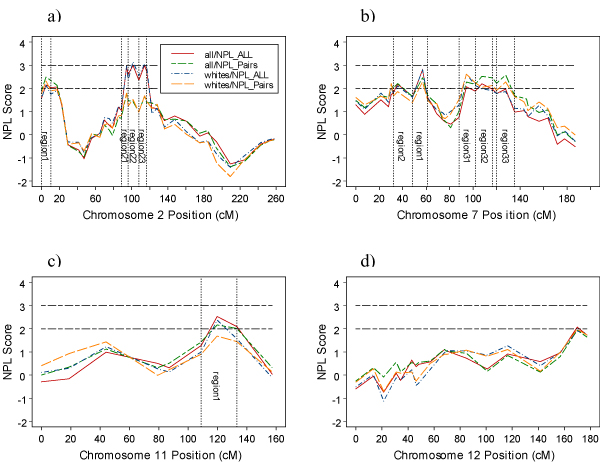
Multipoint NPL score profiles for chromosomes 2, 7, 11, and 12 for all and pairs options using all families and only Whites. Regions used for fine-mapping are also shown.

In the data, 38.8% were affected, 46.2% unaffected, and the rest was unknown. Heritability of 41% with a standard error of 0.17 was estimated for ALDX1 by SOLAR. Chromosome regions showing evidence of linkage with ALDX1 are depicted in Figure 1. The four plots represent NPL score versus map distance based on multipoint linkage analysis using S_all _and S_pair _scoring functions in both the whole sample and Whites only. There was no difference between both samples, therefore we only report the results based on all families. The most significant region is on chromosome 2, which spans marker D2S285 (88.5 cM) to marker D2S1331 (116.5 cM). There are 3 peaks within this wide region. They are labeled as region 21, region 22, and region 23 (Figure 1a). The maximum NPL (S_all_) scores on these regions were 2.92 (*p *= 0.002), 2.99 (*p *= 0.0014), and 2.99 (0.0014) at markers D2S285 (94.2 cM), D2S2109 (101.4 cM), and D2S1790 (114.2 cM), respectively. Suggestive linkage (2.0<NPL<2.8) was also found at different locations on chromosomes 2 (region 1), 7 (region 1, region 2, region 31, region 32, region 33), 11 (region 1), and 12 (D12S2078 (156.8 cM) – D12S392 (177.3 cM)).

### Fine-scale linkage and association mapping

**Figure 2:**
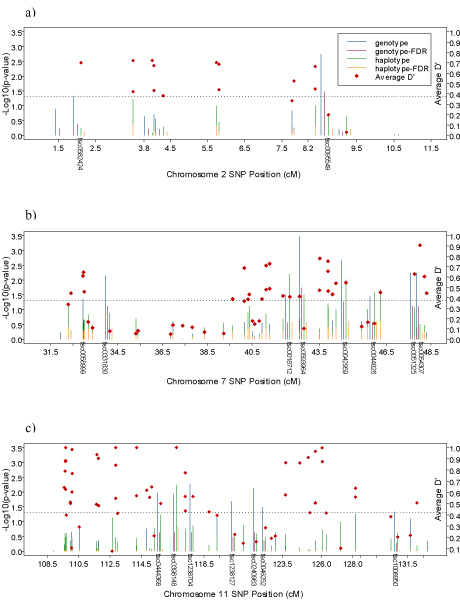
Fine-mapping results from family-based association tests with SNP genotype and SNP haplotypes for region 1 of chromosome 2, 7, and 11. *p*-Values are adjusted for multiple testing by FDR. Mean LD values for each 3-SNPs window are also shown.

The numbers of SNPs used in fine-mapping at different regions given by Figure 1 were 18 (chromosome 2 – region 1, 1 SNP every 223 kb), 23 (chromosome 2 – region 21, 1 SNP every 227 kb), 45 (chromosome 2 – region 22, 1 SNP every 277 kb), 27 (chromosome 2 – region 23, 1 SNP every 701 kb), 54 (chromosome 7 – region 1 (NPY2 region), 1 SNP every 517 kb), 50 (chromosome 7 – region 2, 1 SNP every 205 kb), 60 (chromosome 7 – region 31, 1 SNP every 200 kb), 47 (chromosome 7 – region 32, 1 SNP every 344 kb), 69 (chromosome 7 – region 33, 1 SNP every 300 kb), and 80 (chromosome 11 – region 1, 1 SNP every 203 kb). Most of the single markers, and haplotypes which were found statistically significant at the alpha = 0.05 level became insignificant after FDR adjustment. Region 1 on chromosome 2 and 7 gave us some significant results after adjustment (Figure 2, 2a–b). Figure 2a shows that SNP tsc0095549 (8.55 cM) on chromosome 2 at region 1 remained significant after correction for multiple testing. But the haplotypes around this SNP were not significant even before correction. The SNP tsc0593964 (42.62 cM) on chromosome 7 at region 1 remained significant after correction, but haplotypes of the SNP became insignificant after correction.

We also tested the flanking markers of several candidate genes: ACP1 (tsc0582424, tsc0043640) on chromosome 2, NPY2 (tsc1107783, tsc0058867) and GRM3 (tsc0149750, tsc0040044) on chromosome 7, and DRD2 (tsc0108927, tsc0108926, tsc0108925) on chromosome 11 for association. But we could not find any significant results for either single markers or haplotypes.

## Discussion

In this study, several chromosomal regions on chromosomes 2, 7, 11, and 12 showed evidence for linkage with ALDX1. Some of our results supported previous findings. We were able to confirm some regions previously found for AD on chromosomes 2 and 7. Specially, the region 2 on chromosome 2 and regions 1 and 33 on chromosome 7 are consistent with previous reports [4,5]. We found a maximum NPL score of 2.18 exactly at the NPY2 gene in our region 1 on chromosome 7. We found suggestive evidence of linkage with AD on chromosome 11 around D11S1998 marker, 10.9 cM downstream of DRD2 gene. Fine-scale association mapping was then conducted in these regions using unphased genotype-based and haplotype-based approaches. The power of single marker (genotype-based) association test may suffer because LD information contained in flanking markers is ignored. Haplotype-based methods could be more powerful because they capture available LD information. Surprisingly, our results did not show any advantage of the haplotype-based approach. Indeed, the most significant results in the regions investigated, after correction for multiple testing, were observed with the genotype-based analyses (chromosome 2 – SNP tsc0095549 (8.55 cM) and chr 7 – SNP tsc0593964 (42.62 cM)). Some possible loss of efficiency of the haplotype-based method could partly be explained by the uncertainty in the haplotype inference from genotype due to the unknown phase of the markers. Because SNPs are not very polymorphic markers, phase ambiguity is likely to affect our results. To overcome the problem of phase uncertainty, some studies considered the inferred haplotypes as true haplotypes. This was not done in our study because this strategy could lead to an inflation of type I error and overconfidence in the estimate of the disease locus location [15]. Another explanation is that when there is strong LD between the unobserved causal locus and single marker (genotype), the haplotypes do not carry additional information over genotypes. Indeed, in this situation, a set of marker locus genotypes can easily predict the causal locus while using less degrees of freedom than haplotype-based tests of association [16]. In the chromosomal regions investigated, there were areas with high LD (D' > 0.6). In general, the markers significant with the genotype-based analyses are located within areas with high LD (Figure 2), but this observation is not always consistent. This could suggest that haplotype-based methods might be more advantageous in regions with lower LD. In addition to size of LD, there are other factors that contribute to the relative advantage of each approach. The low densities of SNPs in the studied chromosomal regions also make the comparison of these two approaches difficult, because in this situation both have low power to detect association. It should also be noted that in our analyses, the haplotype-based test of association always involves more degrees of freedom than the genotype-based test, while the same number of tests are conducted with each approach. This could penalize the haplotype-based approach. Therefore, we repeated our analyses by replacing the *p*-value for the global (i.e., multiallelic) haplotype test by the minimum of the *p*-values of the individual biallelic haplotype tests with 1 df. Our results remained unchanged. Also, there might be more efficient methods to perform haplotype-based analyses such as the cladistic analysis [17]. Further studies are needed to better understand the relative advantage of this approach.

Because the regions we investigated included many loci, our findings may be obscured by multiple testing corrections. Therefore, we also investigated several candidate genes (ACP1, NPY2, GRM3, and DRD2) [18-22]. We did not detect any associations with ALDX1, so the comparison of genotype-based and haplotype-based approaches was not possible with COGA data with candidate genes. We also investigated several candidate loci found by genotype-based association (Figure 2a–b) and compared both approaches without correction for multiple testing. In all cases, the genotype-based analysis led to more significant results, whether we used a multiallelic or a biallelic haplotype test of association.

## Conclusion

In conclusion, for this particular population and for the regions investigated, there was no advantage of the haplotype-based analyses over (single-locus) genotype-based analyses. However, caution should be taken when generalizing these results to other chromosomal regions or to other populations. The relative advantage of each approach might be greatly related to the other factors such as the extent of LD, and SNP density in the fine-mapping regions.

## Abbreviations

AD: Alcohol dependence

COGA: Collaborative Study of Genetics of Alcoholism (COGA)

EM: Expectation maximization

FBAT: Family-based association test

FDR: False discovery rate

GAW14: Genetic Analysis Workshop 14

LD: Linkage disequilibrium

NPL: Nonparametric linkage

SNP: Single-nucleotide polymorphism
